# Functional Outcome of Uncemented (Hydroxyapatite Coated) Bipolar Hemiarthroplasty in Fracture Neck of Femur in Elderly

**DOI:** 10.51894/001c.157517

**Published:** 2026-02-12

**Authors:** Dhananjay Sahoo, Suman Sourav Mishra, Anuraag Mohanty, Nikhilesh Das

**Affiliations:** 1 Orthopaedic Surgery Apollo Hospital, Bhubaneswar, India; 2 Orthopaedics Kalinga Institute of Medical Sciences, Bhubaneswar, India; 3 Orthopaedics Peerless Hospital & B. K. ROY Research Center, Kolkata, India

**Keywords:** Femoral neck fracture, Bipolar hemiarthroplasty, Hydroxyapatite coating, Harris Hip Score, Elderly hip fractures, Uncemented stem.

## Abstract

**Background:**

Femoral neck fractures in the elderly are associated with high morbidity and reduced mobility. Uncemented hydroxyapatite-coated bipolar hemiarthroplasty is increasingly used to avoid cement-related complications. This study evaluated postoperative functional outcomes using the Harris Hip Score (HHS).

**Methods:**

A prospective observational study was conducted on 38 elderly patients (≥65 years) with displaced intracapsular femoral neck fractures who underwent uncemented hydroxyapatite-coated bipolar hemiarthroplasty. Functional outcome was assessed using the Harris Hip Score at nine months. Descriptive statistics (mean ± SD) were used.

**Results:**

The mean Harris Hip Score at nine months was 88.4 ± 9.2. Outcomes were classified as excellent in 25 patients (66%), good in 7 (18%), fair in 4 (10%), and poor in 2 (5%). Most patients regained independent ambulation with minimal complications.

**Conclusions:**

Uncemented hydroxyapatite-coated bipolar hemiarthroplasty provides satisfactory early functional outcomes in elderly patients with femoral neck fractures, with high HHS scores and low complication rates over nine months.

## INTRODUCTION

Femoral neck fractures are among the most common and debilitating injuries in the elderly, primarily due to underlying osteoporosis, increased fall risk, and compromised bone quality. Their intracapsular location places the femoral head blood supply at risk, making non-union and avascular necrosis frequent complications.^1,2^ Elderly individuals above 65 years account for the majority of cases, with women disproportionately affected due to postmenopausal bone loss.^3^

The incidence of hip fractures in India has risen substantially with population aging. According to studies, the prevalence of hip fractures in older adults is between 105 and 123 per 100,000 people. As life expectancy rises over the next several decades, this number is expected to rise sharply.^4,5^ The necessity for efficient, low-risk surgical procedures that enable early mobility is highlighted by this increasing burden.

For older patients with displaced intracapsular femoral neck fractures, hemiarthroplasty is still the recommended treatment since it provides a quicker recovery and fewer reoperation rates than internal fixation.^6^ Compared to unipolar designs, bipolar prostheses offer superior functional results and less acetabular erosion due to their dual articulation.^7^ Hydroxyapatite-coated uncemented stems have been introduced to promote biological fixation and avoid complications associated with cement use particularly bone cement implantation syndrome, which can be dangerous in frail geriatric patients.^8–10^

Evidence suggests that hydroxyapatite-coated uncemented implants achieve reliable early stability and allow gradual osseointegration, making them suitable for osteoporotic bone.^11,12^ Several authors have reported favorable mid-term outcomes with such implants in elderly fracture patients, demonstrating good pain relief, functional improvement, and lower intraoperative risk profiles compared to cemented stems.^13–16^

Despite growing adoption, literature from India evaluating functional outcomes of uncemented hydroxyapatite-coated bipolar hemiarthroplasty in elderly patients remains limited. Understanding early postoperative function, complication rates, and rehabilitation patterns is essential for improving clinical decision-making and standardizing care in resource-variable settings.

Therefore, this study aimed to evaluate the nine-month functional outcomes of uncemented hydroxyapatite-coated bipolar hemiarthroplasty in elderly patients with displaced intracapsular femoral neck fractures, using the Harris Hip Score as the primary assessment tool.

## MATERIALS AND METHODS

### Study Design and Setting

This was a prospective observational cohort study conducted at Peerless Hospital and B. K. Roy Research Centre, Kolkata, between September 2020 and June 2022.

### Ethical Approval

The study received approval from the Institutional Scientific Committee (Reference: PHH/SC/NBE/DNB(ORTHO)/3242/2020, approved 26 September 2020). Written informed consent was obtained from all participants or legal guardians prior to enrolment.

### Sampling Method and Recruitment

A convenience sampling approach was used. No formal sample size calculation was performed due to feasibility considerations and the exploratory nature of the study. Forty-five eligible patients were enrolled; seven were excluded during follow-up (three deaths, four lost to follow-up). Thus, 38 patients were included in the final analysis.

### Eligibility Criteria

#### Inclusion criteria

- Age ≥ 65 years
- Displaced intracapsular femoral neck fracture (fresh or delayed)
- Medically fit for anesthesia and surgery (ASA grade I–III; normal ECG; stable cardiopulmonary status; acceptable metabolic profile)
- Able and willing to attend scheduled follow-ups

#### Exclusion criteria

- Cognitive impairment affecting cooperation
- Severe osteoporosis (Singh’s Index Grade I–II)
- Femoral canal deformity or abnormal anatomy
- Associated upper-limb fractures hindering walker-assisted ambulation
- Inability to complete at least nine months of follow-up

### Preoperative Evaluation

**Figure 1.**
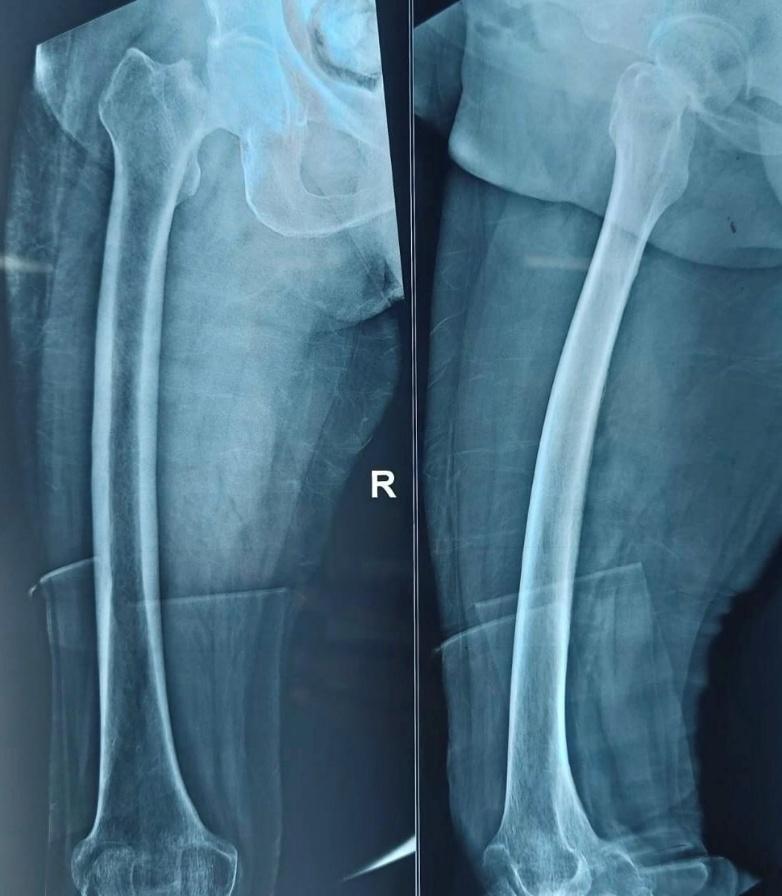
Preoperative anteroposterior pelvic radiograph showing displaced intracapsular fracture of the right femoral neck.

All patients underwent detailed history taking, comorbidity assessment, and clinical examination. Standard radiographs (anteroposterior pelvis and lateral hip views) were obtained. Laboratory evaluations included hemogram, renal and liver function tests, ECG, and chest radiography. Preoperative counseling explained expected outcomes and postoperative rehabilitation. A representative preoperative fracture radiograph is shown in Figure 1.

### Surgical Procedure

All patients underwent uncemented hydroxyapatite-coated bipolar hemiarthroplasty.

To comply with journal guidelines, the detailed operative technique has been moved to Appendix A. A summary is provided here.

**Figure 2.**
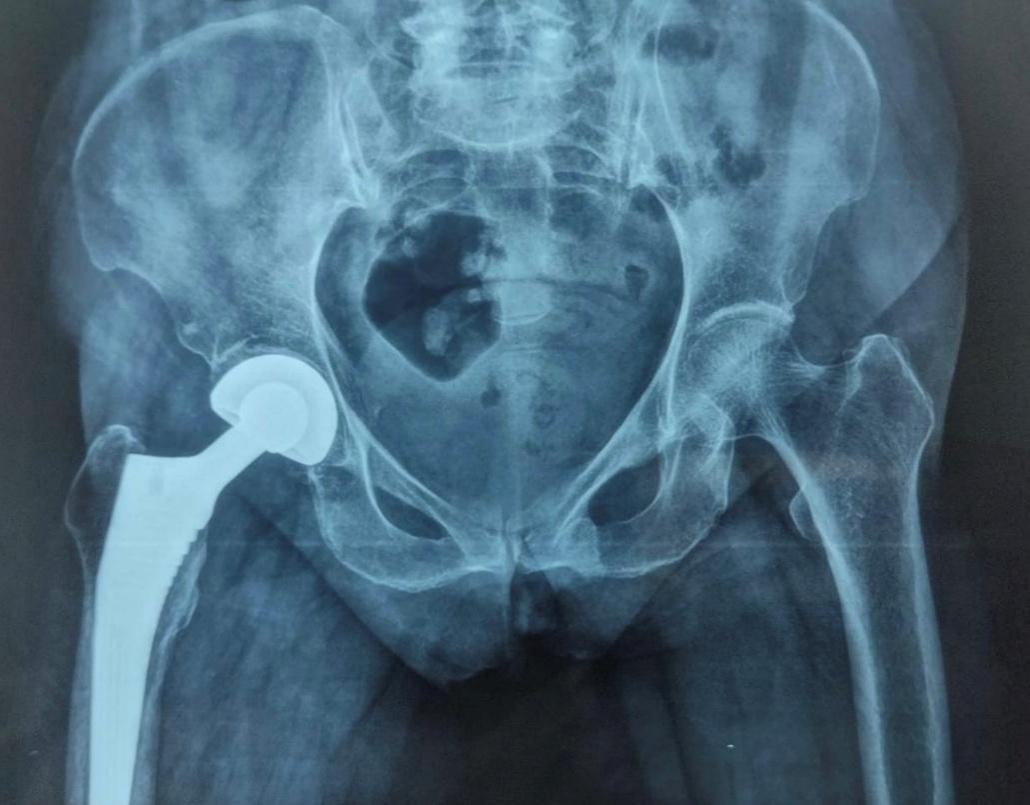
Anteroposterior pelvic radiograph at 9 months postoperatively demonstrating well-positioned uncemented hydroxyapatite-coated bipolar prosthesis.

Surgery was performed under spinal anaesthesia using the modified lateral (Hardinge) approach. After femoral head extraction and canal preparation, a press-fit hydroxyapatite-coated femoral stem and modular bipolar head were implanted. Intraoperative findings, blood loss, implant size, and complications were recorded. A typical postoperative radiograph at nine months is shown in Figure 2.

All procedures were performed by two fellowship-trained orthopedic surgeons, each with 8–10 years of arthroplasty experience.

### Postoperative Care and Rehabilitation Protocol

A standardized institutional rehabilitation pathway was followed:

- **Day 1:** static quadriceps exercises; ankle pumps; bedside sitting
- **Day 2–3:** walker-assisted ambulation as tolerated
- **Week 1–3:** active-assisted hip abduction and ROM exercises
- **Week 3–6:** progressive partial weight bearing
- **Week 6 onward:** gradual transition to full weight bearing based on radiographic stability

**Figure 3.**
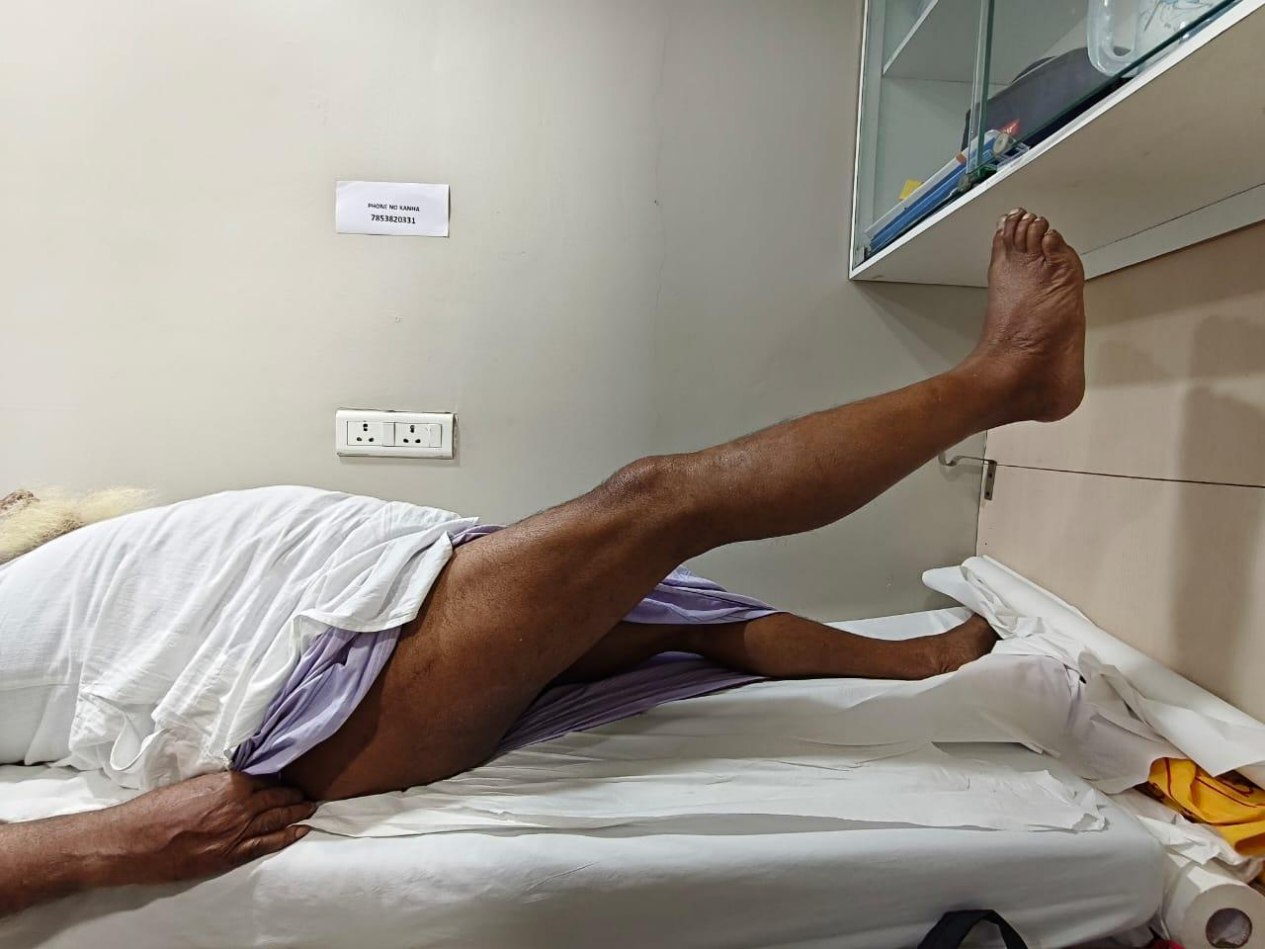
Active straight leg raises at 9 months postoperatively indicating preserved hip flexor strength and neuromuscular control.

**Figure 4.**
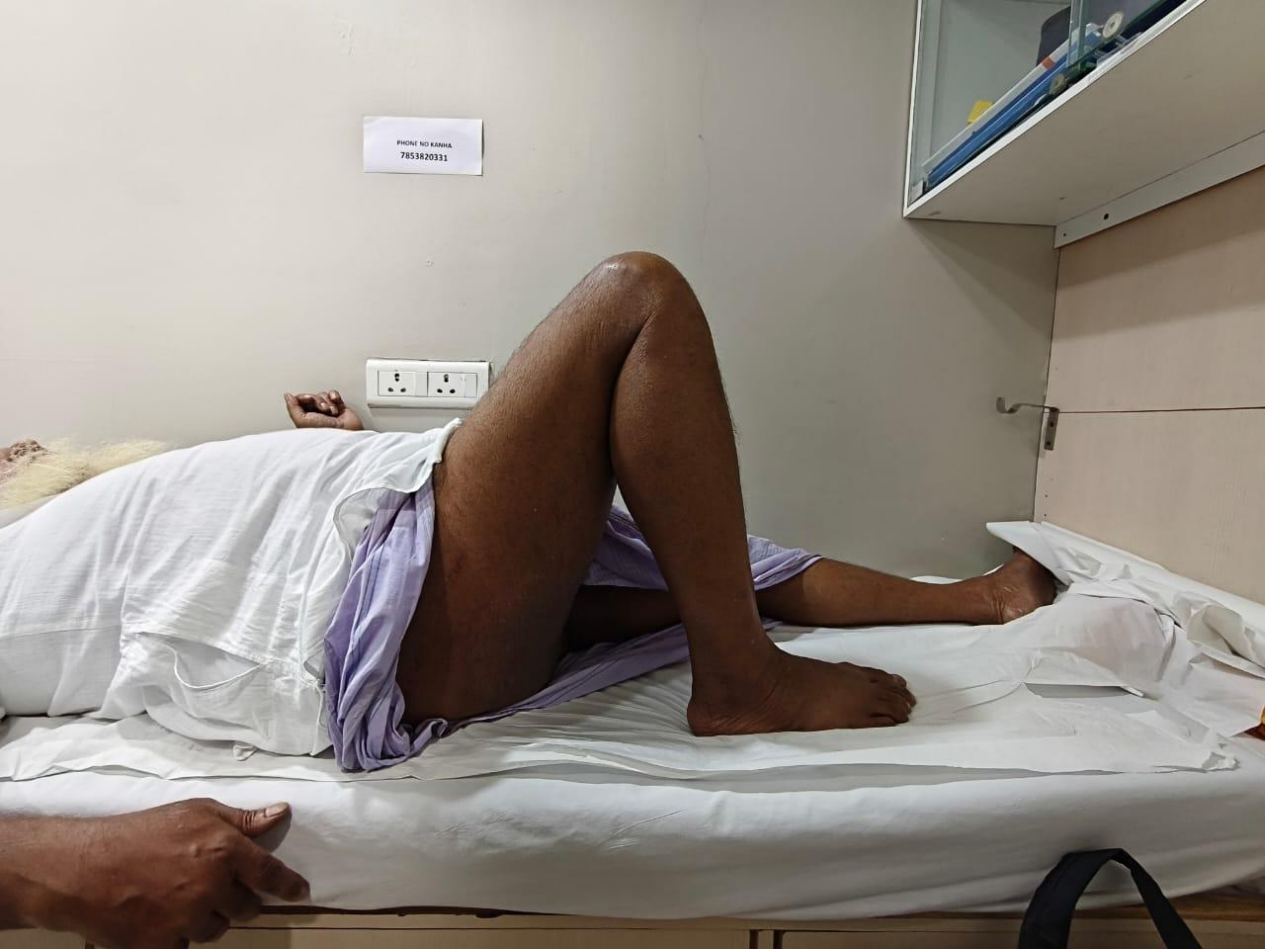
Combined hip and knee flexion at 9 months postoperatively showing maintained joint mobility and functional range.

**Figure 5.**
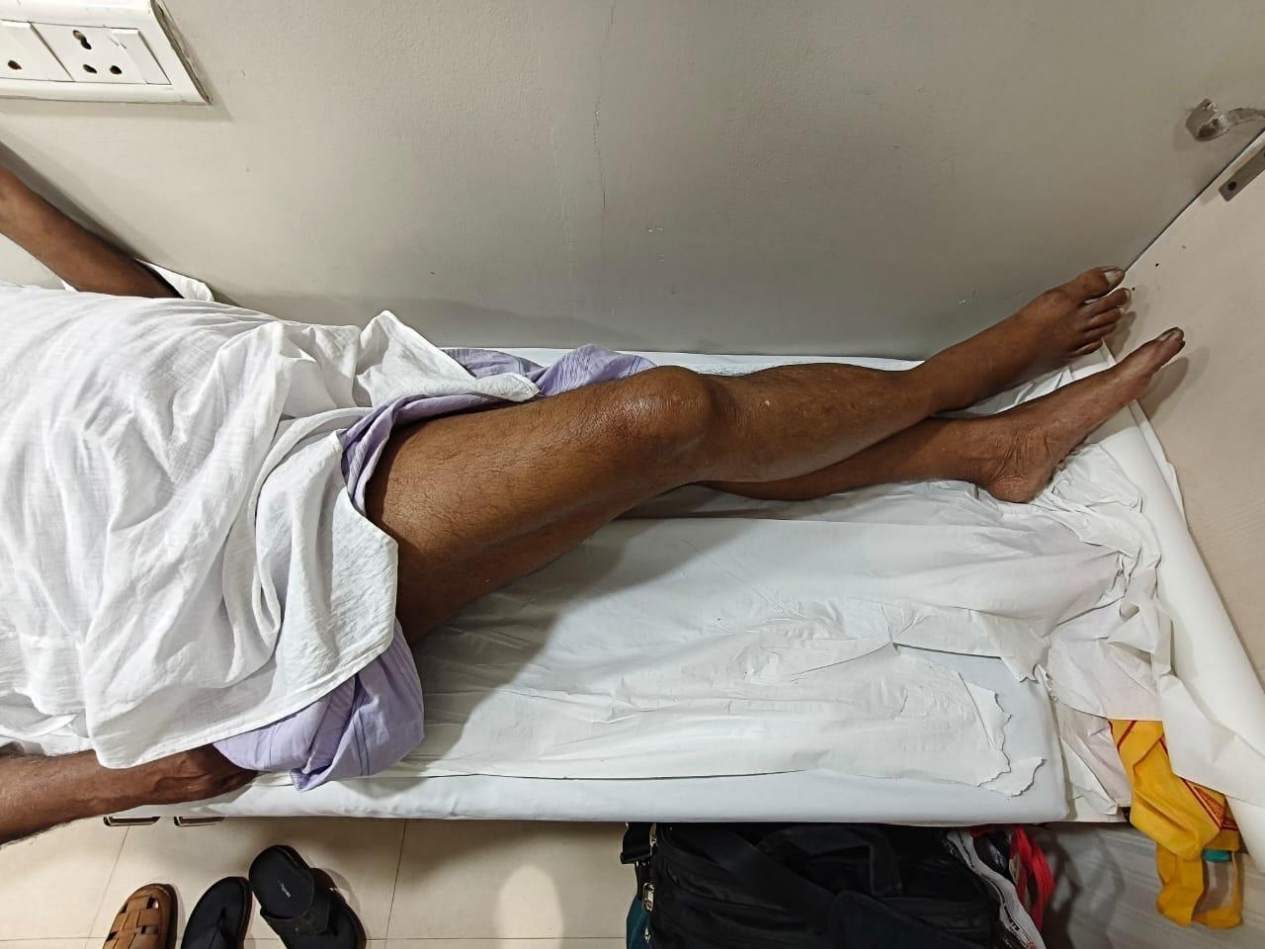
Active hip adduction at 9 months postoperatively indicating recovery of adductor muscle function and medial joint stability.

Hip flexor strength (straight leg lift; Figure 3), combined hip-knee flexion (Figure 4), and adductor strength (Figure 5) were among the functional evaluations.

### Follow-Up Schedule and Outcome Measures

At two, six, twelve, eighteen, and twenty-four weeks as well as nine months, patients were assessed.

The Harris Hip Score (HHS), the main outcome metric, evaluated:

- pain
- gait and support requirement
- functional mobility
- stair climbing
- sitting tolerance
- range of motion

### Radiographic Assessment

Radiographs were evaluated for:

- Stem alignment (neutral/varus/valgus)
- Subsidence (>2 mm vertical migration)
- Calcar resorption
- Radiolucent lines in Gruen zones
- Heterotopic ossification (Brooker classification)
- Periprosthetic fractures
- Evidence of loosening

### Statistical Analysis

A structured proforma was used to enter the data, and IBM Corp.’s SPSS version 29 was used for analysis. Due to the observational design and absence of comparison groups, descriptive statistics such as mean, standard deviation, frequencies, and percentages were employed. Although no inferential statistical tests were carried out, a significance threshold of p < 0.05 was noted for completeness. Under the guidance of a qualified statistician, the research team performed statistical analysis.

## RESULTS

A total of 45 elderly patients with displaced intracapsular femoral neck fractures were enrolled in the study. Three patients expired before completing the minimum follow-up period and four were lost to follow-up; therefore, the final analysis included 38 patients who completed at least nine months of postoperative evaluation.

The mean age of the cohort was 76.2 ± 8.1 years (range: 61–100), with the highest proportion of cases occurring among individuals aged 71–80 years. Females represented 71.1% of the study population, reflecting the higher prevalence of hip fractures in postmenopausal women. Most injuries (89.5%) resulted from domestic falls, and the left hip was slightly more commonly affected (55.3%). The demographic characteristics of the study population are summarized in Table 1.

**Table 1.** Demographic Profile of Patients (n = 38)

Variable	n (%)
Age (years)	
61-70	8 (21.1%)
71-80	17 (44.7%)
≥81	13 (34.2%)
Sex	
Male	11 (28.9%)
Female	27 (71.1%)
Side of Injury	
Left	21 (55.3%)
Right	17 (44.7%)
Mechanism of Injury	
Fall at home	34 (89.5%)
Road traffic accident	4 (10.5%)

Intraoperatively, four patients sustained calcar fractures, all managed successfully with cerclage wiring. Postoperative complications were minor and included two cases of limb length discrepancy (<1 cm), one superficial surgical site infection, and one Grade I bedsore. There were no cases of deep infection, dislocation, thromboembolic events, or periprosthetic fractures. These intraoperative and postoperative complications are detailed in Table 2.

**Table 2.** Intraoperative and Postoperative Complications (n = 38)

**Complication**	**n**	**Management**
Calcar fracture (intraoperative)	4	Cerclage wiring
Limb length discrepancy (<1 cm)	2	Conservative
Superficial surgical site infection	1	Antibiotics
Bedsore (Grade I)	1	Repositioning & local care

Radiographic evaluations throughout follow-up demonstrated satisfactory implant alignment in all patients. Mean stem subsidence was minimal at 0.8 ± 0.4 mm, and no radiographic signs of loosening were observed. Only one patient developed Brooker Grade I heterotopic ossification.

Functional outcomes were assessed using the Harris Hip Score (HHS). At the nine-month follow-up, the mean HHS was 88.4 ± 9.2, indicating generally favourable functional recovery. Most patients reported significant pain relief, with 27 experiencing no pain and nine reporting only mild discomfort. Limp was absent in 33 patients, and 17 patients were able to ambulate independently without external support. Stair climbing was possible for 36 patients, and 24 demonstrated a composite hip range of motion exceeding 210 degrees. Functional recovery details are shown in Table 3.

**Table 3.** Functional Recovery Parameters at 9 Months (n = 38)

**Functional Parameter**	**% of Patients**
Pain-free ambulation	71%
Absence of limp	87%
Independent ambulation without support	45%
Ability to climb stairs	95%
Range of motion > 210°	63%

Based on HHS categories, 25 patients (66%) achieved excellent outcomes, 7 (18%) had good outcomes, 4 (10%) had fair outcomes, and 2 (5%) showed poor outcomes. Thus, 84% of the cohort achieved excellent or good results at nine months. This distribution is presented in Table 4.

**Table 4.** Functional Outcome Based on Harris Hip Score (n = 38)

**Category**	**Score Range**	**n**	**%**
Excellent	90–100	25	66%
Good	80–89	7	18%
Fair	70–79	4	10%
Poor	<70	2	5%

## DISCUSSION

The present study evaluated early functional outcomes following uncemented hydroxyapatite-coated bipolar hemiarthroplasty in elderly patients with displaced intracapsular femoral neck fractures. Most patients demonstrated excellent to good recovery at nine months, reflected in favourable Harris Hip Scores and restoration of mobility, pain relief, and independence. Radiographic assessments also showed stable implant positioning, minimal subsidence, and no loosening, indicating that hydroxyapatite-coated stems provide reliable biological fixation in elderly bone.

These findings align with existing evidence supporting the use of uncemented hydroxyapatite-coated implants in geriatric hip fractures. The osteoconductive qualities of hydroxyapatite, which encourage early osseointegration and improve stem stability, are widely recognized^17–19.^ Similar positive results with uncemented HA-coated stems are reported by studies by Chandran et al. (2012) and Bell et al. (2015), who note improvements in postoperative mobility and a decrease in implant-related problems. These reports are consistent with the significant percentage of patients in our sample who received exceptional or excellent HHS scores.

The decision between cemented and uncemented hemiarthroplasty continues to be a crucial clinical factor. Immediate fixation is possible with cemented stems, however they are linked to bone cement implantation syndrome, which can cause abrupt cardiac impairment in elderly patients who are weak.^17^ Numerous comparative investigations have demonstrated that uncemented stems, particularly those with hydroxyapatite coatings, offer similar functional outcomes without the systemic hazards connected to cement insertion.^18–20^ Our results corroborate this viewpoint: despite the moderate weight-bearing protocol required for uncemented fixation, there were few intraoperative problems, a smooth recovery, and consistently positive functional outcomes.

The study’s demographic pattern, which shows that older women are more likely to have domestic falls, is consistent with the known epidemiology of femur neck fractures.^21^ The burden of fracture in this population is influenced by age-related osteoporosis, diminished proprioception, and elevated fall risk. Our study’s functional recovery patterns, which include high rates of pain-free ambulation and preserved range of motion, support the viability of uncemented hydroxyapatite-coated bipolar hemiarthroplasty, especially in settings with limited resources where reducing postoperative morbidity is crucial.

Only descriptive statistics were employed in the statistical analysis. This decision is in line with the study’s observational methodology, which excluded a comparison group. Because this study is outcome-focused and has a limited sample size, inferential statistical testing would not yield significant new information and would lead to incorrect analytical conclusions. This method is in line with orthopaedic research that looks at functional results in small prospective cohorts.

### Study Limitations

The study is limited in a number of ways. Because of the limited sample size, it is more difficult to make statistically sound conclusions and findings are less generalizable. The lack of a comparator group (like cemented hemiarthroplasty) makes it difficult to compare implant types directly. While the nine-month follow-up time is sufficient for evaluating early functional outcomes, it does not account for longer-term issues such implant survival, acetabular erosion, or late loosening. Because the operating surgeon conducted the radiographic evaluations, observer bias may have been introduced. Furthermore, patients with significant comorbidities, severe osteoporosis, or cognitive impairment were not included, which limited their relevance to the larger senior community. Despite these limitations, the study contributes meaningful early-outcome data supporting the use of uncemented hydroxyapatite-coated bipolar hemiarthroplasty in elderly patients with displaced femoral neck fractures.

## CONCLUSION

For older patients with displaced femur neck fractures, uncemented hydroxyapatite-coated bipolar hemiarthroplasty seems to be a safe and efficient therapeutic choice. Because of the procedure’s low complication rates, stable early fixation, and ease of functional recovery, it is especially appropriate for older patients who may be more vulnerable to cement-related intraoperative occurrences. The hydroxyapatite coating’s biological benefit promotes stable osseointegration in osteoporotic bone, allowing for gradual weight-bearing and adequate mobility following surgery.

From a clinical standpoint, this strategy provides a workable answer in situations where minimizing cardiac strain, cutting down on operating time, and encouraging early recuperation are top concerns. Standardized postoperative care routes can have a major impact on recovery trajectories, making it particularly effective in settings with limited resources.

However, the study’s limited sample size, single-center design, and comparatively short follow-up time limit how broadly these results may be applied. To further understand implant survival, late problems, and overall functional trajectories in this population, longer-term assessment and comparative studies involving cemented implants may be beneficial. Notwithstanding these drawbacks, the current study provides significant early-outcome evidence in favor of the use of uncemented hydroxyapatite-coated bipolar hemiarthroplasty in the treatment of femur neck fractures in older patients.

### Conflict of Interest

None

## APPENDIX A

### SURGICAL TECHNIQUE

The patient was placed in the lateral decubitus position and all procedures were carried out under spinal anesthetic. The method employed was a modified lateral (Hardinge). After making a lazy-J skin incision centered over the greater trochanter, the fascia lata was used to carry out the dissection. The anterior portion of the gluteus medius was separated as necessary to reveal the hip capsule while maintaining posterior tissues for stability following surgery. The fibers were split according to their direction.

After a longitudinal capsulotomy, the femoral head was gently dislocated. Using a corkscrew tool, the head was removed. The femoral neck osteotomy was finished in accordance with the preoperative templating method, around 1-1.5 cm above the lesser trochanter.

A canal finder was used to enter the femoral canal, which was then progressively broached until a stable press-fit was achieved. The resection surface was prepared using a calcar planer to maximize the fit of the uncemented stem against the calcar femorale, which is essential for load transfer.^4^ After that, trial reduction was carried out to evaluate hip stability, femoral offset, and leg length. To restore biomechanics, adjustments were made as needed.

The definitive completely hydroxyapatite-coated uncemented femoral stem was implanted after ideal size and alignment were verified. After attaching the modular bipolar head, the final reduction was carried out. Hip stability was assessed in internal and external rotation, flexion, and extension.

Blood loss, operating time, and implant size were among the intraoperative variables that were noted. During broaching, any calcar cracks were quickly fixed with cerclage wiring. Following haemostasis, the gluteus medius was carefully reconstructed to preserve postoperative abductor integrity, and the incision was closed in layers over a suction drain.^22^ To confirm proper stem alignment and prosthesis position, postoperative radiographs were acquired (Figure 2).
